# The Effect of Muscular Exertion on Proteid Metabolism—The Standard Rations for Working Animals

**Published:** 1881-07

**Authors:** H. P. Ormsby

**Affiliations:** Chemist to Connecticut Agricultural Experiment Station


THE JOURNAL

OF

COMPARATIVE MEDICINE AND SURGERY.

VOL. II. JULY, 1881. No. 3.

### ORIGINAL COMMUNICATIONS.

### ART. XII.—THE EFFECT OF MUSCULAR EXERTION ON PROTEID METABOLISM—THE STANDARD RATIONS FOR WORKING ANIMALS.

THE effect of muscular exertion upon that continual decomposition and oxidation of matter in the body which is conveniently designated by the term *metabolism*, has been the subject of much investigation, and of not a little controversy.

Until within a very short time, however, by far the most abundant and trustworthy experimental evidence has favored the view that muscular exertion, while largely increasing the oxidation of non-nitrogenous matters in the body does not sensibly affect the proteid metabolism. Very diverse conclusions respecting the proximate source of muscular energy have been drawn from this fact, but the fact itself has been almost universally accepted as true. Some more recent investigations, however, seem to indicate that the experiments on which reliance has hitherto been placed may not cover the whole ground. These recent investigations, though not as yet very extended, have yielded some results of much interest, both to the physiologist and to the feeder of working animals, and some account of them may not be without interest to readers of this JOURNAL.

They were made by Kellner,* at the Hohenheim Agricultural Experiment Station in Wurtemberg, on a horse, and differed from earlier experiments chiefly in that the work was continued for a longer time, as well as in being performed on an herbivorous animal. In the experiments of Voit, and of Pettenkofer and Voit, the work was continued only for two or three days, at most; Kellner's experiments included periods of from one to eight weeks, during which the work performed daily (as measured by a dynamometer, constructed for the purpose) was constant. In all the experiments the quantity, chemical composition, and digestibility of the food eaten, were determined in the ordinary way, so that the amount and quality of the nutriment which the animal received were known, while determinations of the urinary nitrogen showed the amount of the proteid metabolism. No determinations of the amounts of carbonic acid and water excreted through lungs and skin could be made, but frequent weighing of the animal supplied their place in a measure.

The first series of experiments was undertaken primarily with a view of testing the effect of muscular exertion on the digestibility of fodder. The daily ration consisted of six kilogrammes (about twelve pounds) of oats, five kilogrammes of hay, and one-fifth kilogramme of straw. It was found that the amount of work performed had practically no influence on the digestibility of the feeding-stuffs employed, and this result was only confirmed by the subsequent trials.

In addition to this object, however, it was undertaken to follow the proteid metabolism in the several periods of the experiment. In the following table is given a summary of the results obtained, each number representing the average per day for the respective period:

---

* London, Jahrbucher, viii., 701, and ix., 651.

SERIES I.

| Period | Work performed. Kilogramme-metres. | Digestible Nitrogen of Food. Grammes. | Nitrogen of Urine.* Grammes. | Volume of Urine. C. C. |
|---|---|---|---|---|
| I. | 475,000 | 134 | 99 | 6,730 |
| II. | 950,000 | 128 | 109 | 6,473 |
| III. | 1,425,000 | 133 | 117 | 8,106 |
| IV. | 950,000 | 126 | 110 | 8,686 |
| V. | 475,000 | 129 | 98 | 9,548 |

These results show plainly that the amount of proteid metabolism, as measured by the ureal nitrogen, varied with the amount of work performed; but before we can conclude that the latter variation was the cause of the former, there are two factors whose influence on the proteid metabolism must be considered.

*First.*—It is a well known fact that an increased excretion of water through the kidneys is accompanied by an increase in the excretion of nitrogen. In these experiments, however, as the table shows, the volume of the urine increased constantly from one period to another, so that this influence cannot well have caused both an increase and a decrease of the ureal nitrogen.

*Second.*—It is a well known fact that muscular exertion makes large demands on the store of fat in the body, and it is equally well established that the fat of the body tends to diminish the proteid metabolism.

If, then, severe work should diminish the amount of fat in the body, we might expect an increase of the proteid metabolism, while on a return to light work fat might be stored up again, and the proteid metabolism diminished. In these experiments, the live weight of the animals varied as follows:

---

* In this first series of experiments the arrangements for collecting the urine were somewhat defective, and some loss of nitrogen probably took place, so that the amount actually excreted was more nearly equal to that digested. The general result in Series 1, however, was fully confirmed by subsequent experiments, more accurately conducted; and this, together with other facts, indicates that the loss of nitrogen was tolerably uniform in all the periods.

| | | |
|---|---|---|
| Average weight in Period | I., | 534.1 kilos. |
| " " " " | II., | 529.5 " |
| " " " " | III., | 522.5 " |
| " " " " | IV., | 508.8 " |
| " " " " | V., | 518.0 " |

Since, as our first table shows, there was no loss of nitrogenous matters by the body, the loss of weight in the first four periods must have been caused chiefly by a destruction of fat, while in the last period a gain took place. As regards the effect on the proteid metabolism, however, we notice that in passing from the third to the fourth period the proteid metabolism fell off, while the loss of fat still continued. Furthermore, it was observed that in passing from a period of more to one of less work (e. g., from III. to IV., or from IV. to V.) the proteid metabolism sank *at once* to about the average for the latter period, while, if the effect were due to an accumulation of fat in the body it would have been gradual. We therefore conclude that the increase in the proteid metabolism in the second, third and fourth periods was a direct result of the greater amount of muscular energy put forth.

Having now considered at some length the first series of experiments, and the conclusions to which it leads, we are prepared to take up the subsequent confirmatory experiments more briefly.

It is a well known fact that during muscular exertion the demand for oxygen in the body is increased, while it also seems to be well established that the maximum amount of oxygen which the system can take up is dependent upon the amount of protein supplied in the food. It is therefore conceivable that in Series I. the food did not supply sufficient protein, that consequently not enough oxygen could be introduced into the body to set free the requisite amount of force by the oxidization of fat, etc., and that this deficiency was made good by the increase of the proteid metabolism. The supposition is not probable, but it is possible.

To test it, a second series of experiments was instituted, in which the protein of the food was considerably increased, while the amount of non-nitrogenous matters was correspondingly diminished, so that the total quantity of nutrients remained nearly the same. A day's ration consisted of 7.5 kilogrammes of hay and 4 kilogrammes of beans. The digestible matters in this and in the previous ration were the following:

| | Protein. Grms. | (Nitrogen.) Grms. | Carbhydrates. Grms. | Fat. Grms. | Total Nutrients. Grms. |
|---|---|---|---|---|---|
| Series I., | 814 | (130) | 4848 | 200 | 5862 |
| Series II., | 1367 | (219) | 3967 | 31 | 5365 |

This series included three periods, in the second of which three times as much work was performed as in the first or third. The following is a summary of the results per day:

SERIES II.

| Period. | Work. Kilogramme-Meters. | Live Weight. Kilos. | Nitrogen Digested. Grms. | Nitrogen of Urine. Grms. | Volume of Urine. C. C. |
|---|---|---|---|---|---|
| I. | 810,000 | 497 | 223 | 199 | 10,168 |
| II. | 2,430,000 | 471 | 217 | 224 | 11,650 |
| III. | 810,000 | 458 | 217 | 200 | 10,528 |

It will be seen that the effect of increased work was the same in this case as in the preceding, viz., to cause an increase in the proteid metabolism. It was likewise observed, as before, that the alteration in the proteid metabolism took place *at once* when the amount of work changed.

The only uncertainty arises from the greater volume of the urine in the second period. It seems doubtful, however, whether this could have caused the observed increase of the ureal nitrogen; and, on the whole, this series confirms the results obtained in Series I.

From the two together we learn, *first*, that the amount of food given in these experiments, while it was sufficient for rest or light work, was not sufficient when hard work was to be done; and, *second*, that under these conditions not only the fat, but also the protein of the body, may be drawn upon to supply the necessary fuel for the lining engine.

Plainly such a loss of protein by the body is very undesirable in practice, and as plainly the way to prevent it is to supply more food. But of what sort shall this food be? Shall we increase the protein of the food, or its non-nitrogenous ingredients? The latter is much the cheaper course, and the facts already in our possession regarding the functions of the protein and of the carbhydrates and fat of the food would lead us to anticipate that it would be also the most effective, but nevertheless much interest attaches to the experimental investigation of this point, which was undertaken in a third series of experiments.

The plan of these experiments was the following: In the first period a light ration, containing beans as one constituent, was given, and a moderate amount of work was performed. In the second period, a part of the beans was replaced by an amount of oats containing the same quantity of digestible protein. In this way the digestible protein of the ration was kept constant, while the non-nitrogenous nutrients were considerably increased, owing to the larger percentage of them contained in the oats. At the same time, the amount of work performed was trebled, and the excretion of nitrogen followed as in previous experiments. In the third period, the amount of work performed was the same as in the first, while the food was continued unchanged from the second period.

The digestibility of the fodder did not correspond exactly with that calculated beforehand, so that the amount of protein actually digested in the second and third periods was a little greater than that in the first. The following were the amounts of the various nutrients digested per day:

| | Protein. Grms. | (Nitrogen.) (Grms.) | Carbhydrates. Grms. | Fat. Grms. | Total Nutrients. Grms. |
|---|---|---|---|---|---|
| Period I., | 1085 | (174) | 5378 | 159 | 6622 |
| Periods II. and III., | 1118 | (179) | 5531 | 245 | 6894 |

In the following table is given, in the same manner as before, the daily averages for each period:

SERIES III.

| Period. | Work. Kilogramme-Meters. | Live Weight. Kilos. | Nitrogen Digested. Grms. | Nitrogen of Urine. Grms. | Volume of Urine. C. C. |
|---|---|---|---|---|---|
| I. | 810,000 | 559 | 174 | 162 | 11,538 |
| II. | 2,430,000 | 541 | 179 | 174 | 10,023 |
| III. | 810,000 | 543 | 179 | 169 | 9,787 |

The great increase in the amount of muscular exertion in the second period was accompanied by only a slight increase in the proteid metabolism, and even this seems to have been due rather to the slightly increased amount of protein in the food than to the work. Moreover, it was observed that the ureal nitrogen was very constant in the second period, being 174 grms. per day in the first half, and 174.8 grms. per day in the second half, while in those experiments in which the work produced a decided increase in the ureal nitrogen this increase was progressive throughout the period.

It appears, then, that the addition to the ration of about 239 grms. of digestible carbhydrates and fat per day sufficed to prevent that increase of the proteid metabolism which we have reason to believe would have taken place without it. By comparing this ration with that of Series II., it will be seen that while it contains a much greater total amount of nutrients, it contains much less protein, the difference being made up by carbhydrates and fat. In Series II., 2,430,000 kilogramme-meters of work caused a decided increase in the proteid metabolism, while in Series III. practically no increase was caused. The difference can only be due to the larger amount of carbhydrates in the second ration.

On the diminution of the work in the third period, the live-weight of the animal increased slightly, and the proteid metabolism sank. Apparently the carbhydrates and fat which were applied to the production of mechanical effects in the second period, being no longer needed for this purpose, produced their well known effect on the proteid metabolism.

It will be seen that these three series of experiments have yielded results of much interest to science. In their consideration, however, certain things must be borne in mind. Investigations of this sort include many sources of error, and require the greatest care in order to give trustworthy results. Furthermore, so many conditions have to be taken account of and controlled, that while an experiment may be scientifically exact in theory, it must in practice be checked by numerous repetitions. That is to say, the method of inquiry which must be adopted in this branch of science is, to a large extent, the statistical method, and a single experiment is, at best, decisive only for the exact conditions under which it was made.

In drawing general conclusions from such experiments as Kellner's, then, we shall remember that they are, after all, but few in number, and shall regard our generalizations as only more or less *probable*, not as certain.

Kellner's results have an important bearing on the much debated question of the proximate source of muscular energy. This source has been found by physiologists of one school in the albuminoids, while those of another school have as positively located it in the non-nitrogenous constituents of the food or of the body. Kellner's experiments certainly seem to show that the organism has a greater power of accommodation in this respect than has been supposed. That the energy of muscular action is furnished by the decomposition of organic matter in the body is now a truism. When the supply of food is sufficient to balance the loss thus occasioned, the body remains *in statu quo*. If the food supply is insufficient, the materials of the body are made to supply the deficiency; and not only the fat, but, according to Kellner's experiments, the albuminoids of the body also, are attacked, so that under these conditions muscular exertion may increase the proteid metabolism.

If, to such a ration, a certain quantity of non-nitrogenous nutrients be added, this increase of the proteid metabolism is prevented, the carbhydrate and fat being decomposed instead of albuminoids. That is to say, under these circumstances the decomposition of non-nitrogenous matters may be a source of muscular energy.

But if Kellner's results are hostile to the views of those who, like Pettenkofer and Voit, see in the albuminoids the sole source of muscular energy, they by no means establish the opinions of those physiologists who look upon the body as the analogue of a heat engine, of which the albuminoids are the machinery, and the carbhydrates and fat the fuel.

In the first place, they show, if they show anything, that albuminoids may be decomposed in the body for the express purpose of supplying muscular energy. Furthermore, they do not show that a working animal does not need more albuminoid food than a resting one. The horse on which these experiments were made required about 400 grammes of albuminoids per day while at rest, to maintain his bodily condition; in these experiments he received from 800 to 1,300 grammes. Whether non-nitrogenous nutrients would have been able to prevent a loss of albuminoids had only 400 grammes per day been fed, may be doubted, but is a question yet to be investigated.

Finally, the results here described do not exclude the hypothesis that the non-nitrogenous matters yield up the potential energy which they contain through the medium of the albuminoids, according to the well known theory of Hermann, which need not be described here.

But these experiments have also an important PRACTICAL BEARING.

They show that when the food of working horses contains a reasonable amount of protein, what is needed when heavier work is to be demanded of the animals, is not more protein, but more carbhydrates and fat. Thus, for the oats of an ordinary ration, we should substitute, not some highly nitrogenous feeding-stuff like beans, but one rich in starch and fat, like maize.

The matter of the use of maize as food for horses is well worthy of attention, particularly for working horses. From a chemical point of view, and particularly in the light of the experiments just described, it is to be highly recommended, and several recent extensive trials of its use, on a large scale, for working horses, are reported to have given favorable results. It is to be hoped that the practicability of the more extensive use of this excellent and cheap grain, as food for horses, will be thoroughly tested.

Unfortunately, neither the recent Hohenheim experiments on the horse, nor the few previously made, have yet furnished sufficient data to render possible the construction of a "feeding standard" for working horses; that is, a statement of the amounts of digestible protein, carbhydrates and fat which the daily ration should contain to produce the desired effect most economically.

A fourth and fifth series of experiments were also performed by Kellner, the results of which may be briefly described. They were in part confirmatory of the previous results, and in part new.

In the fourth series it was attempted to ascertain how much useful work could be produced by the decomposition of a certain quantity of starch in the organism. In the first period a light ration of hay and oats (six kilos of each) was given, and the amount of work was so regulated as just to fall short of the amount required to produce an increase in the proteid metabolism. It may be safely assumed that this amount of work was the maximum amount that could be permanently performed on this ration. One kilogramme of rice starch was then added to the daily ration, and the amount of work increased until it again reached the maximum, as shown by the increase in the proteid metabolism. It was found that with the addition of rice starch about 538,000 kilogramme-meters more work could be performed than without it. Of the rice starch, 613.79 grammes were digested. This quantity of starch, according to the most recent determinations, would yield 2,749,165 gramme-units of heat, equivalent to 1,165,646 kilogramme-meters of work. Accordingly, of the total amount of force liberated in the organism by the oxidation of the starch, about *forty-six per cent.* was available for external work.

A fifth series of experiments was carried out in essentially the same way as the last, but with the substitution of linseed oil for starch. The result was that about *forty-nine per cent.* of the potential energy of the oil was available for external work.

We may say, then, according to these two results, that when to a ration sufficient to maintain the organism we add non-nitrogenous nutrients, about one-half of their potential energy is available for useful work. This result is, of course, only approximate, and is directly applicable only to the conditions of these experiments. Much more investigation is necessary before such a statement can be accepted as proved for all cases, and the interest of these experiments lies chiefly in their suggestiveness.

In conclusion, the writer wishes to call attention to one fact which shows that Kellner's results should be accepted with caution.

It is a well established fact that all the nitrogen which is excreted from the body is excreted either in the urine or fæces, and that in case no gain of flesh has taken place, the total nitrogen of these two is equal to that of the food. It is also tolerably well established that adult animals do not lay in flesh to any considerable extent. Now, in all but one of Kellner's experiments, the nitrogen of the food considerably exceeded that excreted, while at the same time the animal lost weight. These facts tend to raise a suspicion of some defect in the arrangement for collecting the excrements, and, at any rate, warn us not to accept the results too unreservedly until they are confirmed by further experiments.

H. P. ORMSBY, PH. D.,

*Chemist to Connecticut Agricultural Experiment Station.*

